# Breast cancer and tobacco smoke

**DOI:** 10.1038/sj.bjc.6601182

**Published:** 2003-08-26

**Authors:** A J Wells

**Affiliations:** 1 5 Ingleton Circle, Kennett Square, PA 19348, USA

**Sir**,

The Collaborative Group on Hormonal Factors in Breast Cancer has certainly done a disservice regarding the question of the possible connection between tobacco smoke and breast cancer by publishing their recent paper (Collaborative Group, 2002) and concluding that there is no increase in breast cancer risk from smoking cigarettes. They note under Methods that ‘no attention was given to the reported associations of breast cancer with environmental tobacco smoke’. Again, under limitations of these findings in the Discussion section, they say ‘nor has attention been given to the reported effects of environmental exposure to tobacco, as active smoking only has been considered’. However, they make no estimate as to what effect this limitation might have on their findings, nor do they qualify their conclusion in the abstract. Their finding is erroneous because they compared ever-smokers with all never-smokers. Since most never-smokers have had significant passive smoking exposure, and since the passive risk, based on the better studies, is almost as large as the active risk, they, in effect, were comparing exposed with exposed and found no effect.

An estimate of the magnitude of the passive smoking breast cancer risk and its effect on the active smoking risk can be obtained by studying the data in Morabia *et al* (1996) and Johnson *et al* (2000), probably the two best of the 16 or so studies on this issue so far. When these investigators used nonpassively exposed never-smokers as their reference category, Morabia *et al* (1996) found odds ratios of 2.3 (95% confidence interval (CI), 1.5–3.7) for passive smoking and 3.2 (95% CI, 2.1–4.9) by combining odds ratios for three levels of ever active smoking. Johnson *et al* (2000), with a somewhat less comprehensive questionnaire, reported odds ratios for both pre- and postmenopausal women, which, when combined, are 1.43 (95% CI, 1.01–2.02) for passive smoking and 1.7 (95% CI, 1.2–2.2) for active smoking. Using even the lower estimates of Johnson *et al* (2000) to correct the Collaborative Group's active smoking relative risk would have resulted in a substantial, statistically significant, positive risk.
